# Mechanical unfolding of a knotted protein unveils the kinetic and thermodynamic consequences of threading a polypeptide chain

**DOI:** 10.1038/s41598-020-66258-5

**Published:** 2020-06-12

**Authors:** Maira Rivera, Yuxin Hao, Rodrigo A. Maillard, Mauricio Baez

**Affiliations:** 1 Departamento de Bioquímica y Biología Molecular, Facultad de Ciencias Químicas y Farmacéuticas, Universidad de Chile, Santiago, Chile; 2 Department of Chemistry, Georgetown University, Washington, DC 20057 USA

**Keywords:** Single-molecule biophysics, Protein folding

## Abstract

Knots are remarkable topological features in nature. The presence of knots in crystallographic structures of proteins have stimulated considerable research to determine the kinetic and thermodynamic consequences of threading a polypeptide chain. By mechanically manipulating MJ0366, a small single domain protein harboring a shallow trefoil knot, we allow the protein to refold from either the knotted or the unknotted denatured state to characterize the free energy profile associated to both folding pathways. By comparing the stability of the native state with reference to the knotted and unknotted denatured state we find that knotting the polypeptide chain of MJ0366 increase the folding energy barrier in a magnitude close to the energy cost of forming a knot randomly in the denatured state. These results support that a protein knot can be formed during a single cooperative step of folding but occurs at the expenses of a large increment on the free energy barrier.

## Introduction

**Figure 1:**
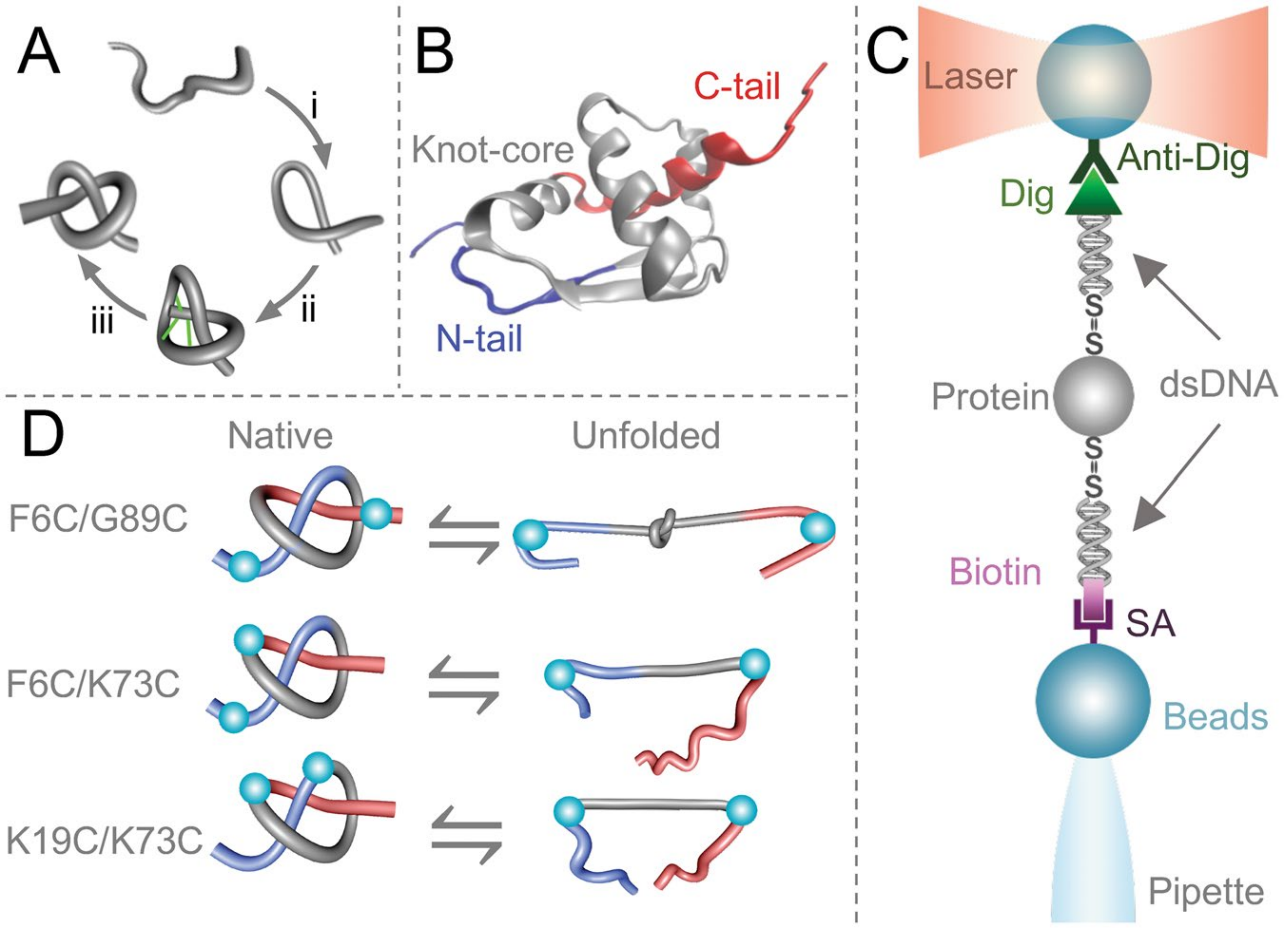
Structure of MJ0366 and experimental design. (**A**) To form a trefoil knot, the polypeptide chain first forms the threading loop (i) that is threaded by one of the protein’s termini (ii). Transient contacts (green lines), formed during threading must break to form the native knot (iii). (**B**) Structure of MJ0366 (PDB 2EFV) showing the knot core of 66 residues (gray), which is threaded by an amino terminal tail (blue) and a carboxy terminal tail (red). The protein representation was generated using VMD version 1.9.4^58^ (https://www.ks.uiuc.edu/Research/vmd/). (**C**) Optical-tweezers experimental setting. A double cysteine mutant is covalently attached to two dsDNA handles. The system is held between two polystyrene beads coated with either streptavidin (SA) or anti-digoxigenin antibodies (Anti-Dig). (**D**) To unfold MJ0366 and preserve the knot in the unfolded state, we applied force from the N- and C-tails of the protein (construct F6C/G89C). To simultaneously unfold the protein and untie the knot, we applied force either from the N-tail and one residue located in the core of the knot (construct F6C/K73C), or from two residues located in the core of the knot (construct K19C/K73C). Cyan beads indicate the pulling points in the construct. The simplified representations of knots were generated by using KnotPlot (http://www.knotplot.com).

Knotted proteins have emerged as a unique class of proteins whose polypeptide chain is self-tied to form the shape of a knot. Over 600 crystallographic structures show the presence of knotted topologies^1^, and many other topological entanglements like slipknots and links has been identified in the protein data bank^2,3^. The folding mechanism of a knotted protein seems difficult because native interactions should create a set of coordinated and timely movements required to thread the polypeptide chain. The constrain resulting from threading limits the number of ways by which the polypeptide reaches the native state, thereby creating a thermodynamic burden for the configurational entropy reduction^4–6^. Early computational studies have suggested that specific weak interactions may be created during early or late steps of threading to help decreasing the topological barrier associated with the formation of a knot^7–9^. These contacts, referred as non-native contacts, occur transiently to guide the threading step, and are eliminated once the native structure of the protein is achieved (Fig. 1A). However, the role of non-native contacts during the folding of knotted proteins remains controversial^6–11^ in part because no experimental evidence has been provided to support the studies of molecular dynamics performed with knotted proteins.

For deeply knotted proteins like α-knotted methyltransferases YibK from *Haemophilus influenzae* and AOTCase from *Xanthomonas campestris*, the explicit contribution of proper non-native interactions to the protein energetics is likely required to form a knot efficiently^7,8^. For these proteins, it is suggested that non-native interactions favor the threading of one end of the chain through a loose loop during early stages of folding. Conversely, for small knotted proteins having shallow knots, such as MJ0366 from *Methanocaldococcus jannaschii*, the thermodynamic bias imposed by the optimization of native contacts could be enough to overcome a large topological barrier of folding^12^. Molecular dynamic simulations performed with native-centric potentials describe that the threading in MJ0366 occurs from the C-terminus via a twisted loop that is stabilized by native interactions^9,12^. Because a twisted loop takes place late in the folding pathway, threading occurs as a diffusive movement in a confined molecular environment. The application of more realistic force fields decrease the folding energy barrier but maintain the overall mechanism described above^9,11,13^. However, these molecular-level descriptions obtained from *in silico* folding simulations have been difficult to verify experimentally.

Several experiments performed with chaotropic agents show the presence of intermediate states during the folding of α/β-knot methyltransferases YbeA and YibK^14,15^, the artificially knotted protein 2ouf-knot^16^, α-haloacid-dehalogenase DehI^17^, and Ubiquitin carboxyl-terminal hydrolase isozymes (UCH) L1^18,19^ and L3^20^. However, the interpretation of such kind of data is difficult because bulk spectroscopic methodologies cannot directly determine whether the folding reaction started from an unknotted or knotted denatured state, or when a knot is formed during their folding mechanism. Moreover, indirect observations indicate that knots seem to be prevalent topologies in the denatured state of deeply knotted proteins^21,22^. To overcome these problems, *in vitro* translation and mechanical unfolding experiments designed specifically to untie the polypeptide chain of YibK, YbeA and UCH-L1 showed that a decrease of the folding rate constant when threading the polypeptide chain is mandatory to reach the native state^23,24^. Besides the intramolecular non-native interactions evolved to overcome the free energy barrier of knotted proteins, it has been proposed that the cellular machinery, like chaperonins and the ribosome, can assist the folding of knotted proteins *in vivo* by promoting the formation of a knot in confined spaces^23,25,26^, by stabilizing key intermediates and establishing new folding routes^10,26–28^, or by modulating the collapse by hydrophobic interactions^29^. These results support that knotted proteins must overcome a topological energy barrier derived from the threading of the polypeptide chain. However, a complete description of the kinetic and thermodynamic consequences related with this process has not been determined experimentally.

Here, we use optical tweezers to study the folding mechanism of MJ0366, a homodimer containing a 3_1_ or trefoil shallow knot into each monomer. Based on the experimental approach reported by Ziegler *et al*. in 2016^24^, we designed three different pulling geometries to either untie or tie the knot in the unfolded state. The mechanical folding or unfolding of MJ0366 was consistent with a simple two-state mechanism for all pulling geometries used in this study. This behavior allowed us to compare the energy cost of forming a knot during the folding of MJ0366 with the energy cost of forming a knot randomly in its unfolded state. We find that knotting the polypeptide chain of MJ0366 strongly increase the energy barrier of folding in a magnitude close to the energy cost of forming a knot randomly in the unfolded state. These observations are discussed in the context mechanism of MJ0366 proposed by *in silico* molecular dynamic and other knotted proteins.

## Results

MJ0366 is a homodimer containing a 3_1_ knot into each monomer (Fig. 1B). The knot core of MJ0366 spans 66 residues (Lys11 to Asn76; gray in Fig. 1B) which is threaded by an N-terminal tail of 10 resides (N-tail; blue in Fig. 1B) and an C-terminal tail of 6 residues (C-tail; red in Fig. 1B)^1,30^. To probe the folding barrier associated with the formation of a knot in MJ0366, we used optical tweezers to mechanically unfold MJ0366 from specific points of its structure (Fig. 1C). This experimental strategy allowed us to compared the free energy profiles of a protein construct designed to preserve the knot in the unfolded state (Fig. 1D, construct F6C/G89C) with protein constructs designed to untie the knot in the denatured state (Fig. 1D, construct F6C/K73C and K19C/K73C). Since MJ0366 has a cysteine at position 81^30^, the three constructs used in this study were characterized in the background of the mutant C81A. Like the wild type protein, all constructs were homodimers in solution (Supplementary Fig. S1A) characterized by indistinguishable unfolding stabilities determined by chemical denaturation under equilibrium conditions (Supplementary Fig. S1B and Table S1).

**Figure 2:**
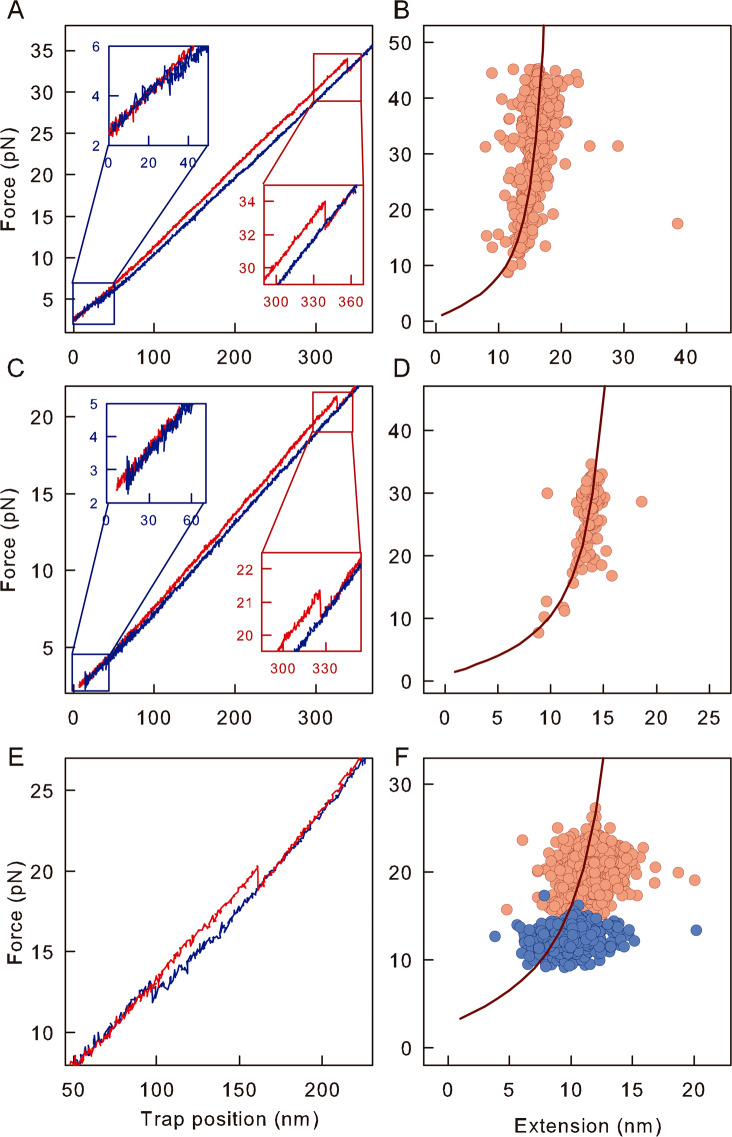
Force-extensions curves of the mechanically unfolding of MJ0366. Unfolding (red) and refolding (blue) trajectories for F6C/K73C (**A**), K19C/K73C (**C**) and F6C/G89C (**E**). Inset: Zoomed-in plot of the unfolding and refolding transitions. WLC analyses of plots of force vs. changes in extension upon unfolding for F6C/K73C (**B**), K19C/K73C (**D**) and F6C/G89C (**F**). Graphics were generated, using SigmaPlot version 10.0 (Systat; https://systatsoftware.com/products/sigmaplot/).

For all protein constructs, force-extension curves obtained at constant pulling velocity display one unfolding transition, indicating the cooperative unfolding of a single MJ0366 subunit irrespectively of the pulling direction (Fig. 2A,C and E). The application of the Worm-Like Chain (WLC) model^31^ to unfolding transitions plots allow us to determine the contour length upon unfolding, Lc (Supplementary Fig. S2). The experimental Lc values for F6C/K73C and K19C/K73C (23 nm and 21 nm, respectively), are in quantitative agreement with the expected molecular extension between the pulling points in the proteins (24 nm and 20 nm, respectively; Fig. 2B,D), indicating complete unfolding during their mechanical perturbation. For F6C/G89C, which is mechanically pulled from the N- and C-tails, we obtained Lc = 23 ± 2 nm (Fig. 2F and Supplementary Fig. S2A), a value that is 7 nm shorter than the one expected for the full-length protein, Lc_theoretical_ = 30.3 nm (84 residues between the pulling points). Similar differences of 4–6 nm have been reported in single molecule studies designed to tight a trefoil (3_1_) knot in the unfolded state^24,32–34^. It is important to highlight that MJ0366 is a homodimer in solution, and no information regarding monomer association or dimer dissociation steps can be extracted from our experiments due to the tethering constrains used to mechanically unfold MJ0366 (Supplementary Fig. S3). However, the value of Lc corroborates the presence of a knot in its denatured state, and directly shows that the interface contacts of the dimer are not required to form a knot in each monomer. In agreement, small conformational changes have been reported for the wild type^35^ or double cysteine dimer when their interface is disrupted by chemical denaturation (Supplementary Fig. S1B).

**Figure 3:**
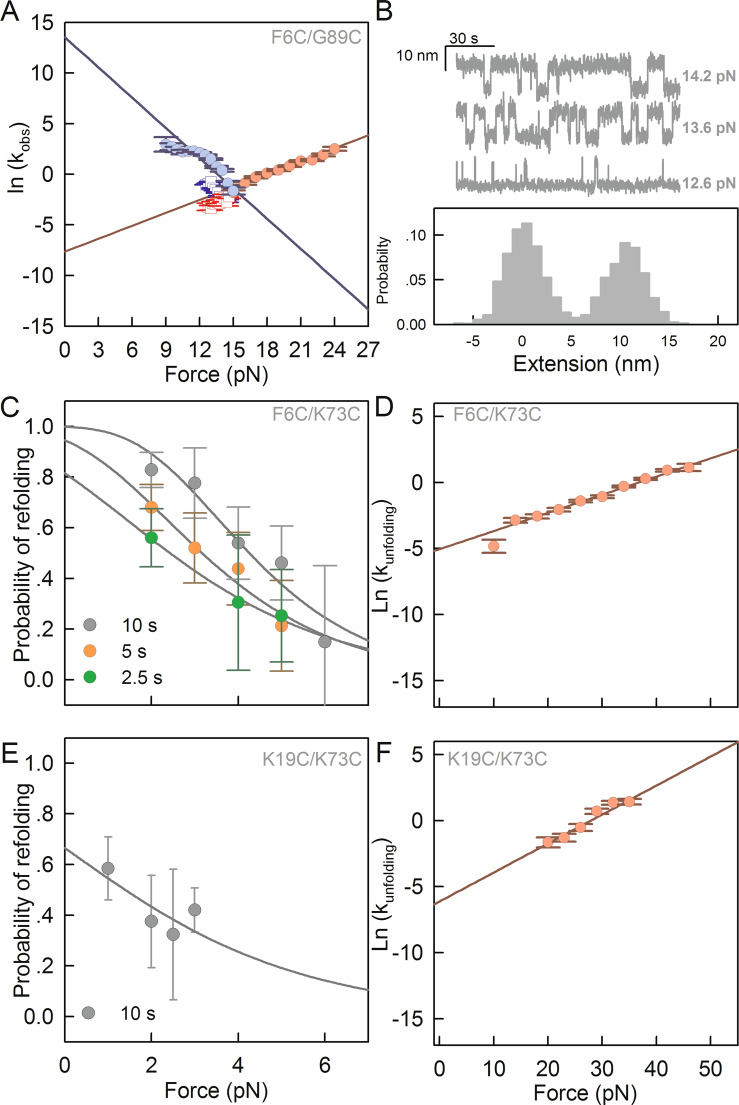
Folding and unfolding kinetics of MJ3066 constructs. (**A**) Force-dependent unfolding (red symbols) and refolding (blue symbols) rate constants for F6C/G89C. The semilogarithmic dependence of rate constants were determined either from the probabilities distribution of forces (circles) or from experiments performed at constant forces (squares). (**B**) Trajectories of extension vs. time for F6C/G89C (Top) and population distribution of folded and unfolded states at F_1/2_ (Bottom). Refolding probability (P_R_) as a function of force for F6C/K73C (**C**) and K19C/K73C (**E**). The waiting times for each curve are indicated in each figure. Force-dependent unfolding rate constants calculated for F6C/K73C (**D**) and K19C/K73C (**C**). Recovered kinetic parameters are shown in Table 1. In experiments performed at constant force (squares in A), error bars represent the fitting error calculated from the exponential fit to the dwell time distribution at each constant force. In experiments performed at constant velocity (circles in A, D and F), error bars correspond to the counting error associated with the frequency of each bin in the force distributions showed in Supplementary Fig. S4. Error bars in C and E correspond to the counting error of the total number of the observed unfolding events. This figure was generated using SigmaPlot version 10.0 (Systat; https://systatsoftware.com/products/sigmaplot/).

The force-dependent rate constants, k(F), were determined by transforming force distributions (Supplementary Fig. S4) using methods developed by Dudko^36^. Dudko *et al*.^37^, also developed an analytical model that incorporates the Kramer’s theory to Bell’s model to describe how the energy barriers and the position of the transition state are affected by force. Experimentally this is reflected in non-linear dependencies of the natural logarithm of the observed kinetic rate constants with force. However, in our case the dependence of the natural logarithm of the kinetic constants with force was linear (Fig. 3A,D and F). Therefore, we fitted our data using the model described by Bell^38^. The Bell model assumes the existence of a single kinetic barrier that the protein needs to energetically overcome to unfold or refold, and that the position of the transition state, (∆x^≠^), does not depend on force. For F6C/G89C, we obtained an unfolding rate constant at zero force (${k}_{U}^{0}$) of 4·10^−4^ s^−1^ and a distance to the transition state from the folded state (∆x^≠^_F→U_) of 1.8 ± 0.5 nm (Fig. 3A, red circles and Table 1). Similarly, we obtained a refolding rate constant at zero force (${k}_{F}^{0}$) of ~10^5^ s^−1^ and a distance from the unfolded state to the transition state (∆x^≠^_U→F_) of 4.1 ± 0.9 nm (Fig. 3A, blue circles and Table 1). We corroborated the results obtained from unfolding and refolding force distributions by conducting time-dependent extension traces at various constant forces (Fig. 3B). At any given constant force, F6C/G89C fluctuates between a compacted or folded state and an extended or unfolded state. The observed change in extension between these two states at 13.6 pN is ~10 nm (Fig. 3B), which matches the end-to-end distance predicted by the WLC at that force (Fig. 2F). Moreover, the unfolding and refolding rate constants determined from time-dependent extension traces (Fig. 3A, squares and S5) agree with those obtained from force distributions (Fig. 3A, circles).

**Table 1:** Kinetic and thermodynamic parameters.

	Unfolding kinetics		Refolding kinetics		Stability	
Constructs	${k}_{U}^{0}$ (s^−1^)^a,d^	∆x^≠^_U_ (nm)	${k}_{F}^{0}$ (s^−1^)	∆x^≠^_F_ (nm)	∆G_kin_ (kcal/mol)^b^	∆G_crooks_ (kcal/mol)
F6C/G89C	4·10^–4^ (3·10^−4^, 1·10^−3^)	1.8 ± 0.5	8·10^5^ (4·10^6^, 3·10^5^)^a,d^	4.1 ± 0.9 ^a,d^	12.7 ± 0.9	13 ± 1
F6C/K73C	6·10^−3^ (2·10^−3^, 2·10^−2^)	0.6 ± 0.1	0.6 ± 0.2^c^	2.2 ± 0.2^c^	2.7 ± 1	—
K19C/K73C	2·10^−3^ (7·10^−4^, 4·10^−3^)	0.9 ± 0.2	0.11 ± 0.04^c^	1.3 ± 0.8^c^	2.4 ± 0.9	—

^(a)^Data obtained using Bell’s kinetic model.

^(b)^∆G_kin_=−RT*Ln(k^0^_U_/k^0^_R_) at 23 °C.

^(c)^Data obtained using refolding probability ${{\rm{P}}}_{{\rm{R}}}=1-{{\rm{e}}}^{(-\varDelta {{\rm{tk}}}_{{\rm{f}}}^{0}\ast {{\rm{e}}}^{(-{\rm{F}}\varDelta {{\rm{x}}}_{{\rm{f}}}^{\ne }/{{\rm{k}}}_{{\rm{B}}}{\rm{T}})})}$.

^(d)^Values a two-sigma confidence interval.

Parameters obtained by using a Bell fitting to the rate constant obtained by constant force experiments or from the force probability distribution.

Having characterized the folding energy profile of F6C/G89C, we used the constructs F6C/K73C and K19C/K73C (Fig. 1D) to investigate the effect of threading the polypeptide chain on folding rate constants and protein stability. Because force-extension curves of the constructs F6C/K73C and K19C/K73C did not reveal clear refolding transitions (Fig. 2A,C, blue traces), we determined refolding rate constants from the probability of refolding^39^, P_R_, at varying refolding forces (2–6 pN) and increasing waiting times (*∆t* = 2.5, 5 and 10 s) (Fig. 3C,E). The fit to the integrated Bell equation^38^ to the values of P_R_ results in ${k}_{F}^{0}$ values in the order of 10^−1^ s^−1^ (Fig. 3C,E and Table 1). Notably, the refolding rate constants for both constructs, which are untied using two different pulling geometries, are six orders of magnitude slower than the folding rate estimated for the construct with the knot in its unfolded state (F6C/G89C, ${k}_{F}^{0}$ ~1·10^5^ s^−1^). These results indicate that the topology of the unfolded state is important for the folding energy barrier which increases significantly when the reaction starts from an unknotted unfolded state. In contrast, the unfolding rates ${k}_{U}^{0}$ range between 10^−3^ s^−1^ for F6C/K73C and K19C/K73C to 10^−4^ s^−1^ for F6C/G89C (Fig. 3A,D,F, and Table 1). A similar trend is observed in the unfolding rate constants extrapolated at zero concentration of guanidinium hydrochloride (GdnHCl), ranging between 10^−2^ s^−1^ for F6C/K73C and K19C/K73C to 10^−3^ s^−1^ for F6C/G89C (Fig. S1C and Supplementary Table S1). It is notable that the relative difference in unfolding rate constants between constructs that untie the knot (F6C/K73C and K19C/K73C) vs. the construct that tightens up the knot (F6C/G89C) is the same irrespectively of the technique used. By using the values of ${k}_{F}^{0}$ and ${k}_{U}^{0}$ at zero force for F6C/K73C and K19C/K73C, we calculated a free energy difference *∆*G_F6C/K73C_ of 2.7 ± 1 kcal/mol and *∆*G_F6C/K73C_ = 2.4 ± 0.9 kcal/mol (Table 1). These values are 10 ± 0.5 kcal/mol lower than the thermodynamic stability estimated for the construct with a knot in its unfolded state determined from the quotient between the refolding and unfolding rate constants or directly by the application of the Crooks fluctuation theorem (F6C/G89C, *∆*G_F6C/G89C_ = 13 ± 1 kcal/mol, supplementary Fig. S6).

**Figure 4:**
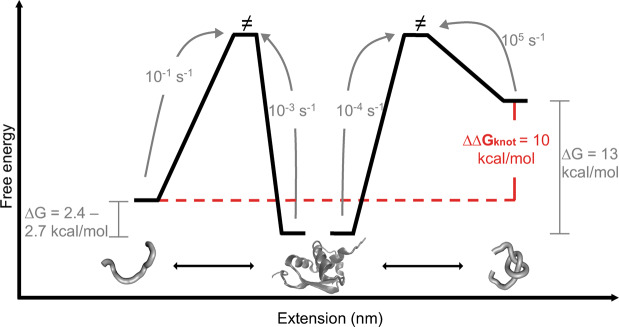
Free energy landscape of MJ0366. The free energy landscape of MJ0366 is constructed by using the thermodynamic and kinetic parameters obtained from this study. When the knot was tightened up in the unfolded state, the calculated stability is ~13 kcal/mol. In contrast, the free energy difference decreases to 2.4–2.7 kcal/mol when refolding begins from the unknotted denatured state. The value of ∆∆G_knot_ correspond to the energy cost to form a knot in the denatured state. The arrows represent the folding and unfolding rate constants determined from the denatured or native state respectively. The protein representations were produced with VMD version 1.9.4^58^ (https://www.ks.uiuc.edu/Research/vmd/). The images of the unfolded states were generated using KnotPlot (http://www.knotplot.com).

Figure 4 summarizes the thermodynamic and kinetic characterization of MJ0366. It should be noted that the energy of the native states, for the different constructs, is equivalent in the free energy profile of Fig. 4. This normalization is supported by two reasons; the unfolding kinetics is about 10^−3^ – 10^−4^ s^−1^ for the three constructs (Table 1) indicating that the free energy barrier for the mechanical unfolding of MJ0366 does not depend on the pulling geometry and, chemical denaturation experiments indicate that all constructs have a similar stability, ranging from 5.2 to 6.0 kcal/mol (Supplementary Fig. S1B, Table S1) with similar unfolding rate constants extrapolated at zero molar of GdnHCl (Supplementary Fig. S1C and Table S1). Therefore, we surmise that the energy difference between F6C/G89C and either F6C/K73C or F19C/K73C is originated by the destabilization of the unfolded state, the state where the knot is tightened or untied in the setup of the optical tweezers. As shown in Fig. 4, the normalization of the native state’s energy for the different protein constructs reveals that the 3_1_ knot increases the energy of the unfolded state by 10 ± 0.5 kcal/mol (∆∆G_knot_). Since ∆∆G_knot_ is calculated between two unfolded states on the same protein, the energy cost is mainly entropic and comparable to the entropic cost of knotting a polypeptide. In other words, the value of ∆∆G_knot_ likely represents the energy cost paid by the unfolded state to form a knot just by chance. This value is similar to the knotting cost calculated for the artificial knotted protein Arc-L1-Arc^34^.

Besides the thermodynamic stabilization of the denatured state by a knot, the kinetic analysis represented in Fig. 4 suggests two folding pathways for MJ0366; a slow-folding pathway involving the formation of a knot (${k}_{F}^{0}$^*unknotted*^ = 0.1 s^−1^, right side of Fig. 4) and a fast-folding pathway starting from a denatured state already knotted (${k}_{F}^{0}$ ^*knotted*^ ~1·10^5^ s^−1^, left side of Fig. 4). Therefore, although the protein can fold very fast from a knotted unfolded state, that fast pathway is unlikely to occur because the large thermodynamic bias towards the unknotted denatured state (∆∆G_knot_ = 7.7 to 9 kcal/mol kcal/mol). The slow-folding rate ${k}_{F}^{0}$ ^*unknotted*^ = 0.1 s^−1^ seems unusually slow for a small single domain protein of 92 residues like MJ0366, while the fast-folding rate ${k}_{F}^{0}$ ^*knotted*^ ~10^5^ s^−1^ is similar to the single-chain version of the repressor ARC, a small monomeric artificial homologue of MJ0366 devoid of a knot in its hydrophobic core^40^. ARC folds very fast at about 1·10^4^ s^−1^ ^34,40^. Therefore, the formation of a trefoil knot during the folding of MJ0366 represent a large constrain that strongly increase the folding barrier (∆∆G^≠^_knot_) by 8.4–9.4 kcal/mol (∆∆G^≠^_knot_ = −RT*Ln(${k}_{F}^{0}$ ^*unknotted*^*/*${k}_{F}^{0}$ ^*knotted*^*)*.

## Discussion

The large energy barrier increment associated with the threading in MJ0366 contrasts with the effect reported for the folding barrier of more complex proteins like YibK, YbeA and UCH-L1. It was observed in *in vitro* translation experiments that the folding kinetics of YibK and YbeA is reduced 0.7 to 10 times compared to the rates determined starting from their knotted unfolded state^14,23^. Similarly, mechanical unfolding experiments showed that the formation of a 5_2_ knot in the structure of UCH-L1 decreases the folding rate constant at zero force by 10-fold^24^. In terms of free energy, the formation of a knot increases the value of ∆∆G^≠^_knot_ between 0.7 and 2 kcal/mol. In comparison, the formation of a shallow knot in MJ0366 involves a large energy cost of *∆∆*G^≠^_knot_ of 8.4–9.4 kcal/mol. At first glance, this result seems intriguing because YibK, YbeA and UCH-L1 require the formation of deep and complex knots during folding, respectively, and therefore are expected to have similar or higher energy barriers when compared with a shallow 3_1_ knotted protein like MJ0366. However, this comparison assumes that deep and complex knotted proteins have evolved similar strategies to overcome the intrinsic topological barrier of proteins with shallow and simpler knots.

Knotted proteins like YibK, YbeA and UCH-L1 display a broad repertoire of folding intermediates in chemical and mechanical denaturation studies^14,15,18^. The presence of intermediates suggests that the overall energy cost of forming a knot could be paid by a progressive stabilization of partially folded structures with the drawback of the formation of off-pathway intermediates, like in the case of mechanical and chemical perturbations in UCH-L1^18,24^. Additionally, since both methyltransferases need to thread a large extension of a chain and UCH-L1 forms a complex 5_2_ knot, it is plausible that the contribution of non-native contacts effectively reduces their folding energy barriers. In support of this interpretation, folding simulations performed with a native centric potential revealed that the fraction of successful folding events is rather low for self-tying deeply knotted proteins, but it is significantly increased by the addition of specific non-native contributions^7–9^. Thus, it is possible that mutations on critical residues required for non-native interactions could increase the free energy barrier associated with the threading step, or alternatively increase the population of off-pathway and aggregating-prone intermediates^18^.

In contrast to observing several folding intermediates^14–20,24^, our data for MJ0366 are consistent, with a two-state folding mechanism. For example, for all constructs we always observed a single distribution of both unfolding forces (Supplementary Fig. S4A) and molecular extension (Fig. 3B) when the protein is refolded from the knotted denatured state. A similar two-state folding mechanism is deduced when refolding occurs from the unknotted denatured state. In this case, the unfolding transitions produce a single force and distance distributions irrespective of the waiting time used to refold the protein (Fig. 3C and E)). The absence of detectable intermediates is consistent with the cooperative unfolding of each monomer of MJ0366 reported under equilibrium conditions^35^. Therefore, our experiments provide an opportunity to compare the thermodynamic stability with the values estimated from chemical denaturation experiments monitored with circular dichroism (Supplementary Fig. S1). When the kinetic and thermodynamic parameters derived from bulk and single molecule experiments are compared, there is a good agreement between the constructs designed to unite the knot in the denatured state of MJ0366 (F6C/K73C and K19C/K73C) than the construct designed to tie the knot in the denatured state of MJ0366 (K6C/K89C). For example, although the chemical stabilities for F6C/K73C and K19C/K73C differ by 2 kcal/mol with respect to their mechanical stabilities, this difference is about 8 kcal/mol for the construct designed to tie the knot (Table 1 and Supplementary Table S1). Considering that the stability calculated by chemical denaturation experiments performed under equilibrium conditions is not only similar among all constructs (Supplementary Table S1) but also similar to the stability calculated for the constructs designed to untie the knot with optical tweezers (F6C/K73C and K19C/K73C, Table 1), we surmise that chemical denaturation effectively unties the knot in the denatured state of MJ0366.

There are several molecular dynamic simulations that explain the mechanism of threading of MJ0366^9,11,12,25,41–43^. In general, the application of native centric potential support the formation of an on-pathway intermediate, guided by native interactions, needed to organize the structure for a further high energy step required to form the knot^9,12^. Experimentally, our single molecule experiments and those obtained by chemical denaturation^35^ were unable to detect an intermediate, but its formation cannot be discarded since its structure is predicted to be unstable and very short-lived^12^. Nevertheless, a key aspect of these mechanisms is the ability of MJ0366 to form a knot without an explicit energetic contribution of non-native contacts, albeit at the expense of a large energy barrier^9,12^. This barrier, entropic in nature, arises from a limited number of accessible folding routes leading to a competition between threading and the packing of the polypeptide chain^12^. This feature could explain the large energy cost associated with the formation of a knot during the folding of MJ0366 (*∆∆*G^≠^_knot_ = 8.4–9.4 kcal/mol) which is comparable to the entropic cost to form a knot in the unfolded state of MJ0366 (∆∆G_knot_ = 10 ± 0.5 kcal/mol). Structure-based molecular dynamic simulations on MJ0366 predict a peak in the free energy as a function of global parameter such as number of native contacts (Q). This peak correlates with threading of the polypeptide chain^12^. In agreement with the computational evidence, we observed experimentally a strong kinetic effect on the folding rate constants between unknotted and knotted unfolded proteins. Thus, if the contribution of non-native contacts for the folding of MJ0366 (for example by stabilization of the transition state) was significant, then it should be expected a lower value of ∆∆G_knot_ with respect to the unguided formation of knot in the denatured state of MJ0366. Therefore, we postulate that the formation of a trefoil knot in MJ0366 is an unavoidable topological constrain, whose energy cost is transferred to the folding barrier without the assistance of non-native contacts.

Considering that a knot in the unfolded state of MJ0366 accelerates its folding by several orders of magnitude, chaperone encapsulation seems a plausible mechanism to increase its apparent folding rate constant^25,43^. Experiments with chaperonins like GroEL-GroES show a 20–40-fold acceleration of knotting of some proteins like YibK and YbeA relative to the folding initiated from their already knotted denatured state, respectively^23^. Although a molecular mechanism has not been yet determined, molecular dynamic simulations carried out in cylinders that mimic chaperonins suggest that a confined environment may enhance self-tying by increasing the probability of forming a knot randomly and by promoting alternative folding routes^25,43^. Nevertheless, simulations performed with native centric potential show that a rapid packing or compaction of MJ0366 increase its folding barrier by increasing the formation of topological trap^12^. In this respect, a recent study performed with lattice and off-lattice models of knotted proteins, the authors explore the role of intermolecular hydrophobic interactions established early during the chaperonin cycle^29^. They find that strong hydrophobic interactions between protein and chaperonin cage counterbalance a (bad) side effect of steric confinement while moderate hydrophobic intermolecular interactions with the cage lead to a significant enhancement of knotting probability in relation to bulk conditions while simultaneously moderating the effect of steric confinement.

The process of co-translational folding also has been proposed to aid during the formation of knots in proteins^10,27,41^. However, by using a minimalist model of the ribosome exit tunnel, Chwastyk and Cieplak reported subtle effects on the folding mechanism of MJ0366^41^. Nevertheless, this model does not consider the potential formation of specific interactions between a nascent chain and the ribosome surface, which have been shown to be a critical aspect to explain the productive folding of a deeply knotted protein^7,8^. Specifically, Dabrowski-Tumanski *et al*. using coarse-grained molecular dynamics proposed that threading of a nascent chain could occurs co-translationally through a twisted loop stabilized by specific interactions near of the ribosomal exit tunnel^27^. Since weak interactions between the ribosome and nascent chains has been described experimentally^44^, it is plausible that intermolecular interactions –rather than non-native interactions– could contribute to form a knot for proteins with intricate and complex topologies like MJ0366.

## Methods

### MJ0366 constructs design

Three different pulling geometries were used in this study, one to conserve the knot upon mechanical denaturation and two to untie it. To maintain the knot in the unfolded state, a construct was design by placing cysteines at the N-tail (F6C) and the C-tail (G89C). However, as MJ0366 has a cysteine at position 81, a C81A mutation was added as well. The other two pulling geometries were designed to untie the knot upon pulling based on the approach developed by Sułkowska *et al*.^45^. To untie the knot by the C-tail, one cysteine was placed at the N-tail (F6C) and the other one at the core of the knot (K73C). Here, to theoretically assure the untying of the protein the condition p_1_ < 2p_2_ – N must be met, where p_1_ is 6, p_2_ is 73 and N is the total number of residues. For the construct F6C/K73C, the condition to untie the knot upon stretching is accomplished (6 < 49). To untie the knot by pulling from the core of the knot now p_1_ is 19 and p_2_ is 73. If there are no interactions that interrupt the unknotting of the polypeptide chain, the velocity of untying is the same in both ends. Therefore, each pulling site will slide 27 residues upon stretching. Since p_1–27_ = −8 and p_2_ + 27 = 100, theoretically the construct K19C/K73C will untie upon mechanical denaturation.

### Gene cloning, protein expression and purification

The MJ0366 coding sequence (gene symbol MJ_RS01930) was cloned into the pET21d (+) (Novagen) vector with a 6xHis tag at the C-terminal of the proteins. Proteins were overexpressed and purified as reported in Ramírez *et al*.^46^. To eliminate the affinity tag, the TEV protease cleavage site ENLYFQG was added between the C-terminal and the His tag. The dimeric state of the mutants was confirmed by size exclusion chromatography using a Superdex S75 column (GE Healthcare).

### Determination of MJ0366 stability and unfolding kinetics by chemical denaturation using circular dichroism spectroscopy

The equilibrium denaturation and renaturation curves were determined in the presence of GdnHCl between 0 and 6 M. Protein samples were incubated at several concentrations of GdnHCl for 1 hr at 23 °C in 30 mM Tris-HCl pH 8, 6 mM Na_2_PO_4_ pH 8, 500 mM NaCl, 1.5% glycerol and 1 mM DTT. Under these conditions the stability curves obtained with circular dichroism (CD) were superimpose. Far UV CD spectra were acquired in a Jasco-1500 dichrograph, using a protein concentration of 10 µM in a 1 mm cell. The folding stability curve was analyzed as indicated previously^35^, considering the unfolding of the monomer of MJ0366 through a two-state model of unfolding. For the unfolding kinetics experiments, the native protein at 100 µM was manually diluted to 10 µM in 30 mM Tris-HCl pH 8, 6 mM Na_2_PO_4_ pH 8, 500 mM NaCl, 1.5% glycerol and 1 mM DTT 10 µM at several concentrations of GdnHCl. The change in secondary structure was measured at 222 nm. The observed kinetic traces were fitted to a single exponential decay using SigmaPlot version10.0 (Systat) and, the dependence of the observed kinetic constants with GdnHCl was fitted to a linear regression to determine m values and the kinetic constant at 0 M of chaotropic.

### DNA handles attachment to proteins

Proteins were concentrated to 3–5 mg/ml, incubated overnight with 50 mM DTT at 4 °C and purified by gel filtration in 50 mM Na_2_PO_4_ pH 8 and 500 mM NaCl. The eluted protein was incubated with a 70-fold excess of 2,2’-Dithiodipyridine (DTDP) during 15 to 60 min. The excess of DTDP was removed by consecutively using two Micro Bio-Spin (Bio-Rad) previously equilibrated in 50 mM Na_2_PO_4_ pH 8 and 500 mM NaCl. The DNA handle attachment was performed as described previously by Hao *et al*.^47^. Briefly, the activated proteins were incubated with of 5′-Tiolmodifed dsOligos of ~33 bp. The formation of the double labeled protein-ds-Oligos complex was evaluated by electrophoresis in a 15% Tris-Glycine polyacrylamide gel (Supplementary Fig. S7A) and purified by electroelution. The electric field for the electroelution was 3 V/cm for 1 hr, followed by one minute of 5 V/cm with the poles inverted. The purified protein was dialyzed against 50 mM Na_2_PO_4_ pH 8 and 500 mM NaCl for 2 hrs. Next, the protein-dsOligos complex was ligated to dsDNA handles of 333 and 356 bp chemically modified with biotin or digoxigenin, respectively. The ligation was monitored by electrophoresis in a 6% TBE polyacrylamide gel (Supplementary Fig. S7B).

### Optical tweezers experiments

Experiments were performed using a MiniTweezers device^48^. The protein samples were manipulated in presence of 50 mM Na_2_PO_4_ pH 8 and 500 mM NaCl. The protein-DNA complexes were incubated with 3.1 µm anti-dig coated beads for 15 min at 20 °C and injected to the microfluidic chamber. As shown in Fig. 1C, a 3.1 µm bead was trapped by the laser beam and a second 2.1 µm bead coated with streptavidin was held by suction in a micropipette tip. To catch a molecule between the two beads, the 3.1 µm bead was moved towards the 2.1 µm bead, by moving the optical trap closer to the pipette tip. The presence of a single DNA-protein complex was evaluated by the determination of the overstretching distance of the dsDNA handles at ~65 pN^49,50^, which corresponded to ~95% of the theoretical total length expected for a DNA handle of 716 bp.

### Determination of kinetic and thermodynamic folding parameters

The force dependence of unfolding and refolding rate constants were extracted from the force distribution using the model developed by Dudko *et al*.^36,37^. As the dependence of force and the natural logarithm of rate constants was linear, we fitted this data using the model described by Bell^38^. As indicated in the Supplementary Fig. S4, the experimental distributions of unfolding and refolding forces were well predicted by the kinetics parameter calculated at zero force (Table 1). We determined the error of the unfolding rate constants, as the counting error of each bin in the force distributions (See Supplementary Fig. S4). This error was calculated as the inverse of the root square of the total events (frequency) contained in each bin ($Counting\,error=1/\sqrt{frequency\,per\,bin}).$

In the case of F6C/K73C and K19C/K73C the refolding transitions were not apparent form the force-extension trajectories. Therefore, the values of *k*_0_ and Δ*x*^≠^ were evaluated indirectly through the refolding probability (P_R_) calculated by the chance to observe an unfolding rip during the stretching cycle as indicated in the main text^39^. Data using three different waiting times (1, 5 and 10 s) was only collected with the mutant F6C/K73C, which allowed us to perform a global fit to the integrated form of Bell equation^39^. In the case of the mutant K19C/K73C data was obtained at only 10 s of waiting time.

The kinetic parameters of the F6C/G89C mutant, were also determined from experiments at constant force under equilibrium conditions. In this case, the temporal variation of the molecular fluctuation, were performed by using the electronic feedback system to keep the force constant during the change of the trap position^51^. The response time of the electronic feedback is 1 ms, which is faster than the molecular interconversion dwell times observed in the interval of forces explored which are in the order of seconds. The hopping traces where obtained for 11 molecules of F6C/G89C between 12.6 to 14.6 pN every 0.2 pN during of 2–5 min at each force (Fig. 3B). F6C/G89C hops only between two states; a compact (native) and extended (unfolded) state. The dwell times in each state were determined using the pCLAMP software version 10.6 (Molecular Devices)^52^ and the unfolding and the refolding rate constants at different forces were determined from the exponential distributions of dwell time of the native and unfolded states respectively (Supplementary Fig. S5). The values of Δ*x*^≠^ and *k*_0_ were determined from the semi-logarithmic form of the Bell equation^38^.

The thermodynamic free energy of stability between the native and unfolded states were determined from the quotient between the refolding and unfolding rate constants ($\varDelta G=-\,RTLn\left(\frac{{k}_{0}^{U}}{{k}_{0}^{R}}\right)$) or directly by the application of the Crooks fluctuation theorem^53,54^ for the mutant F6C/G89C. The calculation of reversible work from constant velocity pulling experiments was determined as described previously by Bustamante *et al*.^34^.

### Calculating contour length

The experimental values of contour length (Lc) were determined from the distributions of ratios between the experimental extensions changes (Δ*x*_exp_) with respect to the theoretical extension (Δ*x*_theoretical_) expected to occur at identical forces as described previously^34^. The values of Δ*x*_theoretical_ at several forces were determined by numerical integration of the WLC model^55,56^, which includes the distance between the cysteines in the native state (Fd). For the mutants F6C/G89C, F6C/K73C and K19C/K73C the value of Lc_theoretical_ are 30.3 nm; 24.4 nm and 19.8 nm respectively; and the value of Fd are 4.1 nm; 1.9 nm and 1.9 nm, respectively. The values of Lc_theoretical_ were calculated considering a persistence length of 0.65 nm and a Lc of 0.365 nm per residues^57^. The Lc at each particular rupture force was determined as Lc_theoretical_ • ((Δx_exp_+Fd)/Δx_theoretical_). The average Lc was obtained by performing a Gaussian fit to the Lc distribution histogram for the mutants here reported (Fig. S2).

## Supplementary information

Supplementary information.
