# Supplementary material for: Mechanical unfolding of a knotted protein unveils the kinetic and thermodynamic consequences of threading a polypeptide chain
Source: Sci Rep. 2020 Jun 12;10:9562. doi: 10.1038/s41598-020-66258-5 (PMC7292828; doi:10.1038/s41598-020-66258-5)
Supplement: Supplementary file 1 — Supplementary information. [file 41598_2020_66258_MOESM1_ESM.pdf]

Supplementary Information for

Mechanical unfolding of a knotted protein unveils the kinetic and thermodynamic consequences of threading a polypeptide chain

Maira Rivera, Yuxin Hao, Rodrigo Maillard and Mauricio Baez

This PDF file includes:

Supplementary Figs. S1 to S6
Table S1
References for SI citations

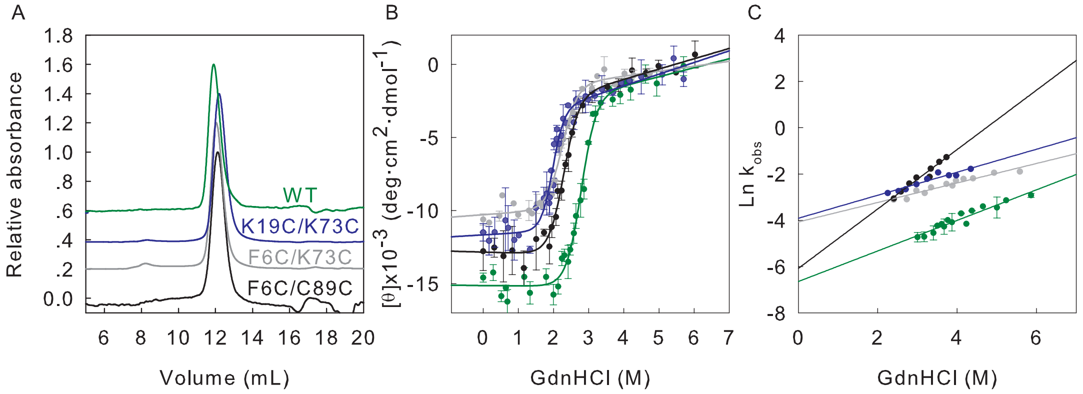

Figure S1. **Hydrodynamic properties and chemical stability of double cysteine mutants of MJ0366. (**A) Chromatograms of double cysteine mutants determined by size exclusion chromatography at 280 nm. **(**B) Equilibrium stability curves of the double cysteine mutants of MJ0366. The change of secondary structure was followed by circular dichroism at λ = 222 nm and plotted as molar ellipticity as function of guanidine hydrochloride concentration (GdnHCl). Same color code in both panels: F6C/G89C (black), F6C/K73C (grey), K19C/K73C (blue) and wild type MJ0366 (green). The continuous lines represent the fitting to a two-state folding model (N ⇌ U). The experimental molecular weight and parameters of stability are indicated in the Table S1. (C) Observed unfolding rate constants as function of GdnHCl. The change in secondary structure in function of time was followed by circular dichroism at 222 nm. The continuous lines correspond to a linear fit of the observed unfolding rate constants for F6C/G89C (black), F6C/K73C (grey), K19C/G89C (blue) and wild type MJ0366 (green). The Graphics and analysis were performed using SigmaPlot version 10.0 (Systat; https://systatsoftware.com/products/sigmaplot/).

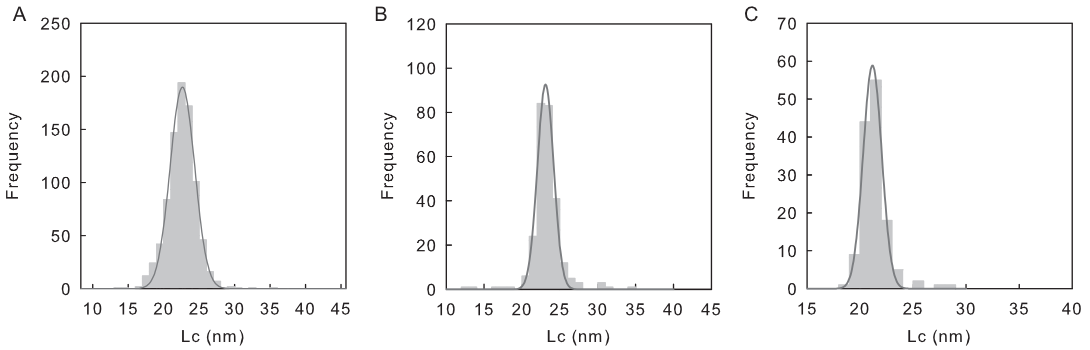

Figure S2. Contour length distribution of double cysteine mutants of MJ0366. The distribution of experimental contour length (Lc) determined from the molecular extensions is indicated in methods. The average values of the experimental Lc values for F6C/G89C (A), F6C/K73C (B) and K19C/K73C (C) were 23 ± 2, 23 ± 1 and 21.2 ± 0.9 nm, respectively. The errors are calculated by the gaussian fit to the frequency distribution data (continuous line). Graphics and analysis were performed using SigmaPlot version 10.0 (Systat; https://systatsoftware.com/products/sigmaplot/).

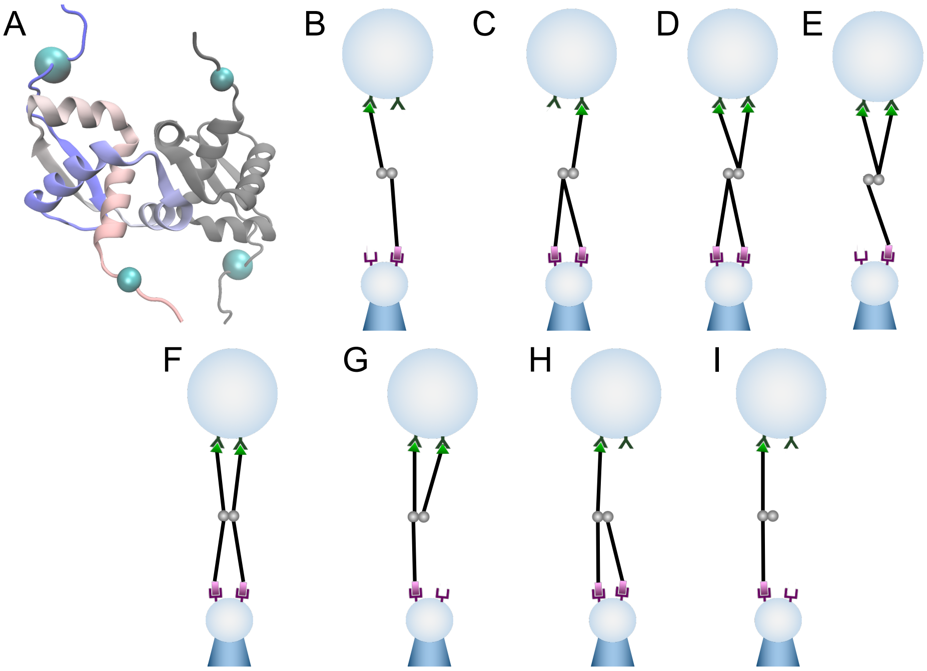

Figure S3. Non-redundant dimer-DNA stoichiometries and geometries compatibles with a MJ0366 dimer placed between a bead coated with anti-dig and other bead coated with streptavidin. A. Dimeric form of MJ0366, which one monomer is in gray while the other is in a color scale blue to red following from N- to C- terminal. The cysteines of the F6C/G89C mutant are highlighted in cyan. There are two scenarios by means a dimer (gray spheres) can be placed between a bead coated with anti-dig and other bead coated with streptavidin. In the first scenario (upper panel), one type of DNA handle is attached to one monomer (for example Dig, green triangle) and other type of DNA handle is attached to the second monomer (For example Bio, purple square). This situation given rise to four different geometries with different stoichiometries of covalent modification (B to E). All these combinations are incompatible with our results because the first mechanical unfolding of each monomer or the mechanical disruption of the dimeric interphase will break the connection between the beads. Therefore, it is impossible or very unlikely to obtain hundreds of folding and refolding cycles for single molecule as we observe. In the second scenario (F to I) two types of DNA handles (Bio and Dig) can be attached to one monomer and thus many cycles of unfolding and refolding can be obtained with a single molecule. However, the case of F, two transitions of unfolding or refolding should be observed since both monomers are connected to both beads. Also, the overstretching of the DNA handle should occur over 60 pN by the presence of two pair of DNA handles. However, we always observe a single unfolding or refolding transition and each molecule reported in the manuscript is validated by the overstretching or the observation of a single event of detaching. In the cases G and H, the adjacent monomer will remain attached to only one bead while in the case I the adjacent monomer will diffuse into the chamber since the structure of each monomer is required to form the dimeric interface. Therefore, subsequent unfolding and refolding events must come from a single monomer attached to two DNA handles. This situation is compatible with our results since the Lc calculated for all constructs is comparable with the size of a monomer. Also, unfolding and refolding forces present a single distribution supporting a two-state mechanism of unfolding. Additionally, from the point of view of the structure, the dimeric interphase is not intertwined, and each monomer represents a well-defined domain. Indeed, experiments of chemical denaturation indicates that each monomer undergoes a little conformational change upon dissociation, and both unfolds independently accordingly with their m-values[1]. These results and analysis justify the application of a simple two-state mechanism of unfolding for the observed mechanical transitions. The protein representation in A was generated with VMD version 1.9.4[2] (https://www.ks.uiuc.edu/Research/vmd/).

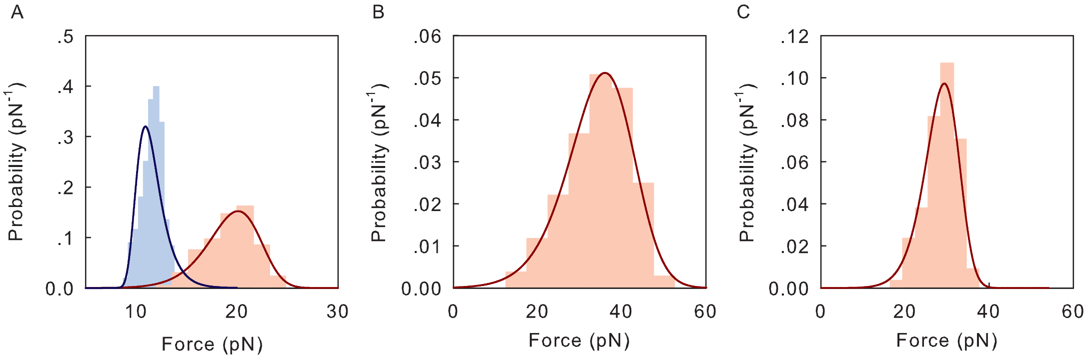

Figure S4. Normalized unfolding probability force distributions of MJ0366. The unfolding (red) and refolding (blue) force probability distributions of the mutants F6C/G89C (A) and unfolding distribution (red) for F6C/K73C (B) and K19C/K73 (C). The lines represent the simulations to describe the distribution of unfolding forces using the Bell[3] equation and the kinetics parameter indicated in the Table 1 of the main text. The graphics were performed using SigmaPlot version 10.0 (Systat; https://systatsoftware.com/products/sigmaplot/).

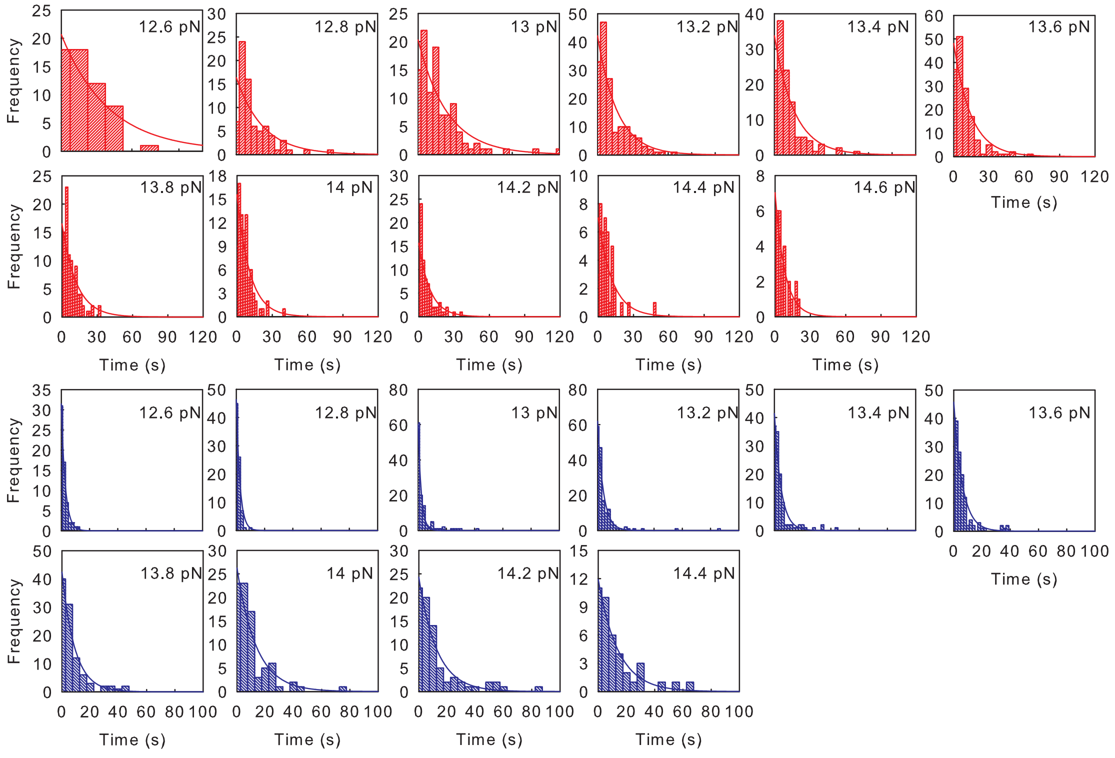

Figure S5. **Dwell time distributions of states determined at constant force for mutant F6C/G89C.** Native and unfolded dwell time distributions at different forces are represented in red and blue respectively. The constant force applied is indicated in each plot. The lines correspond to the fitting of the experimental data to single exponential decay. Graphics and analysis were performed using SigmaPlot version 10.0 (Systat; https://systatsoftware.com/products/sigmaplot/).

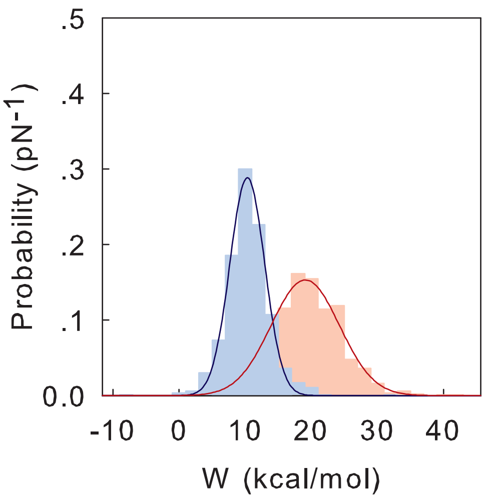

Figure S6. **Work probability distribution for F6C/G89C**. Normalized unfolding (red) and refolding (blue) probability distributions. ΔG of 13 ± 1 kcal/mol was calculated from the intersection between both distributions. Graphics and analysis were performed using SigmaPlot version 10.0 (Systat; https://systatsoftware.com/products/sigmaplot/).

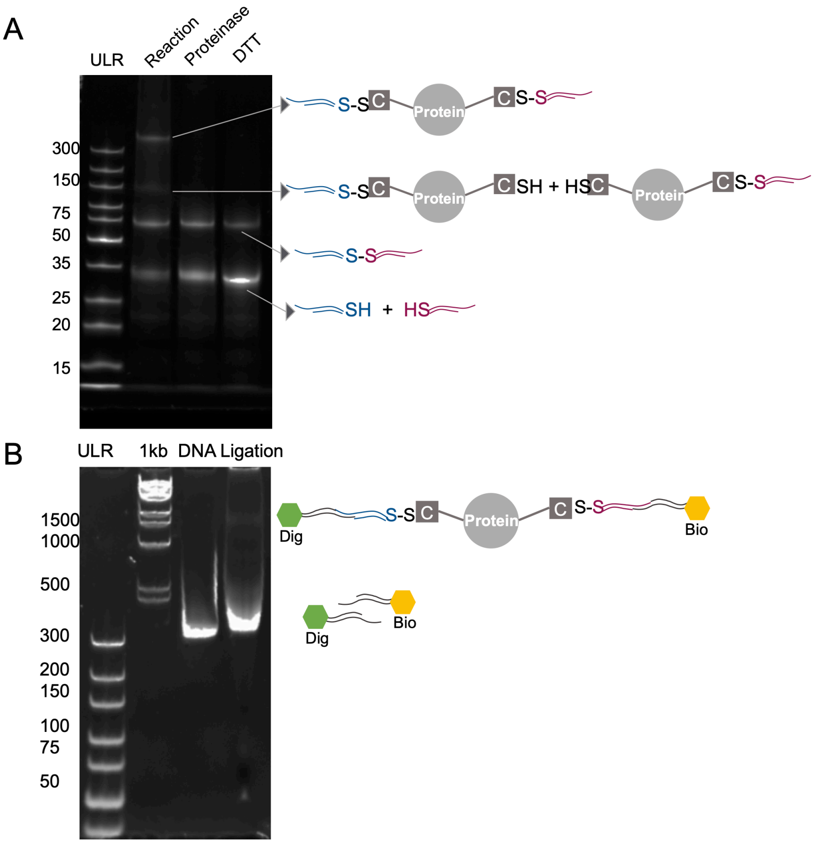

Figure S7. **Covalent attachment of DNA handles to double cysteine mutants.** 5′-Tiolmodifed dsOligos were attached to double cysteine mutants via disulfide bond (A). In the gel, from left to the right: molecular weight marker (ULR), overnight incubation of the protein with dsOligos in the absence (Reaction) or presence of 1 µg proteinase K (Proteinase) or with 50 mM of dithiothreitol (DTT). The dsOligos have a molecular weight of ~33 bp. The protein-oligo chimera of ~300 bp was purified and ligated with long (370 bp) dsDNA handles modified with digoxigenin (green square) and biotin (orange circle) (B). The 1500 bp band corresponds to the dsOligo-protein with correctly attached dsDNA handles. The chemical modification was determined by gel electrophoresis stained with GelRed in a 15% Tris-Glycine native polyacrylamide gel (A) or in 6% polyacrylamide gel in TBE (B). The molecular species observed in the gels are indicated in the figure based on their expected molecular weight. Experimental details are given in material and methods. Gel analysis was performed with Image Lab software version 6.0.1 (Bio-Rad; https://www.bio-rad.com/es-es/product/image-lab-software?ID=KRE6P5E8Z) by using the auto scale option for brightness and contrast adjustment.

	SEC	Stability		Unfolding kinetics	
Constructs	Molecular mass (kDa)	ΔG (kcal/mol)	m (kcal/mol*M)	k_U^0 (s^{-1})	m_U (kcal/mol*M)
Wild type	20.8	8.2 ± 0.6	3.1 ± 0.2	$1.3 \cdot 10^{-3} \pm 3 \cdot 10^{-4}$	0.39 ± 0.03
F6C/G89C	19.1	6.0 ± 0.7	2.7 ± 0.3	$2.3 \cdot 10^{-3} \pm 8 \cdot 10^{-4}$	0.76 ± 0.06
F6C/K73C	19.4	6 ± 1	2.7 ± 0.6	$1.7 \cdot 10^{-2} \pm 2 \cdot 10^{-3}$	0.24 ± 0.02
K19C/K73C	17.9	5.2 ± 0.9	2.7 ± 0.4	$2.0 \cdot 10^{-2} \pm 5 \cdot 10^{-3}$	0.29 ± 0.04

Table S1. Apparent molecular weight and chemical protein stability of double cysteine mutants

REFERENCES

1. Wang, I., Chen, S.-Y. & Hsu, S.-T. D. Unraveling the Folding Mechanism of the Smallest Knotted Protein, MJ0366. *J. Phys. Chem. B* **119**, 4359–4370 (2015).
2. Humphrey, W., Dalke, A. & Schulten, K. VMD: Visual molecular dynamics. *J. Mol. Graph.* **14**, 33–38 (1996).
3. Bell, G. I., Dembo, M. & Bongrand, P. Cell adhesion. Competition between nonspecific repulsion and specific bonding. *Biophys. J.* **45**, 1051–64 (1984).
